# Spatial and Temporal Analysis of Impacts of Hurricane Florence on Criteria Air Pollutants and Air Toxics in Eastern North Carolina

**DOI:** 10.3390/ijerph19031757

**Published:** 2022-02-03

**Authors:** Sharmila Bhandari, Gaston Casillas, Noor A. Aly, Rui Zhu, Galen Newman, Fred A. Wright, Anthony Miller, Gabriela Adler, Ivan Rusyn, Weihsueh A. Chiu

**Affiliations:** 1 Department of Veterinary Integrative Biosciences, Texas A&M University, College Station, TX 77843, USA; sharmilatbhandari@gmail.com (S.B.); casillas.gaston@gmail.com (G.C.); naly@cvm.tamu.edu (N.A.A.); irusyn@cvm.tamu.edu (I.R.); 2 Interdisciplinary Faculty of Toxicology, Texas A&M University, College Station, TX 77843, USA; 3 Department of Landscape Architecture and Urban Planning, Texas A&M University, College Station, TX 77844, USA; zr1991@tamu.edu (R.Z.); gnewman@arch.tamu.edu (G.N.); 4 Bioinformatics Research Center, Departments of Statistics and Biological Sciences, North Carolina State University, Raleigh, CA 27695, USA; fred_wright@ncsu.edu; 5 Entanglement Technologies, San Bruno, CA 94066, USA; amiller@entanglementtech.com; 6 Breezometer, Haifa 3303124, Israel; gabrielaa@breezometer.com

**Keywords:** air pollution, geospatial analyses, criteria pollutants, volatile organic compounds

## Abstract

Natural and anthropogenic disasters are associated with air quality concerns due to the potential redistribution of pollutants in the environment. Our objective was to conduct a spatiotemporal analysis of air concentrations of benzene, toluene, ethylbenzne, and xylene (BTEX) and criteria air pollutants in North Carolina during and after Hurricane Florence. Three sampling campaigns were carried out immediately after the storm (September 2018) and at four-month intervals. BTEX were measured along major roads. Concurrent criteria air pollutant concentrations were predicted from modeling. Correlation between air pollutants and possible point sources was conducted using spatial regression. Exceedances of ambient air criteria were observed for benzene (in all sampling periods) and PM2.5 (mostly immediately after Florence). For both, there was an association between higher concentrations and fueling stations, particularly immediately after Florence. For other pollutants, concentrations were generally below levels of regulatory concern. Through characterization of air quality under both disaster and “normal” conditions, this study demonstrates spatial and temporal variation in air pollutants. We found that only benzene and PM2.5 were present at levels of potential concern, and there were localized increases immediately after the hurricane. These substances warrant particular attention in future disaster response research (DR2) investigations.

## 1. Introduction

The increase in the frequency and intensity of hurricanes and typhoons is one of the most notable signs of climate change [1]. While the monetary losses due to destruction of property are among the most notable concerns regarding natural disasters [2], the potential for immediate and delayed human health effects from environmental mobilization of contaminants is also widely acknowledged [1,3]. Increasing attention is being devoted to the potential for natural disasters to affect the release, fate, and transport of air pollutants in the environment [4]. Air pollution includes both criteria air pollutants regulated by national ambient air quality standards (e.g., ozone, particulate matter, lead, nitrogen dioxide, carbon monoxide, and sulfur dioxide) and air toxics, such as volatile organic compounds (VOCs).

The adverse health effects of criteria air pollutants are well established and the scientific evidence for ambient air quality standards are periodically updated by various regulatory bodies [5]. The associations between climate change, criteria air pollutants and human health are of concern because of the likely exacerbation of their formation/releases through the increased frequency/severity of extreme events, such as hurricanes, wild fires, and heat waves [6].

Air toxics, such as VOCs, are released in significant amounts during petroleum refining and chemical manufacturing, as well as by motor vehicles [7]. For example, BTEX (benzene, toluene, ethylbenzene and xylene) is an important class of VOCs, is a major component of traffic-related air pollution, and is discharged by burning fuels such as gasoline, wood, coal or natural gas and during chemical manufacturing [8]. The acute and chronic health effects of BTEX, including neurotoxicity, hematotoxicity, and carcinogenicity, are well established [7]. BTEX also play an important role in the overall air quality as they participate in photochemical reactions, producing ozone and secondary organic aerosols, effects that are exacerbated by climate change [9]. During disasters, either industrial accidents or emergency shutdowns of petroleum refineries in anticipation of hurricanes, major releases of hazardous air toxics have been documented [10,11]. Other common sources of exposure to hazardous air toxics that are related to natural disasters are indoor mold and mildew, which may be potent sources of allergens, and increased use of consumer cleaning products, which may be sources of VOCs [12,13].

Few studies have been conducted to determine whether regional air quality may be adversely impacted by natural disasters in ways that are unrelated to refinery operations, such as the effects on traffic patterns and/or massive releases of air pollutants from other point sources that may be impacted. For example, gasoline spills and leaks from petroleum storage facilities due to structural damage were noted as among the major sources of air toxic releases after Hurricane Harvey [14]. After Hurricane Maria, the widespread use of power generators was associated with localized increases in emissions of air toxics [15]. After Hurricane Katrina, air quality concerns were primarily associated with indoor exposures to VOCs [16].

In all recent large-scale disasters, detailed evaluation of air quality was severely hindered by the fact that most stationary monitors were inoperable, either due to the loss of power or preventative shutdowns [14,16]. Therefore, an opportunity exists to use mobile analytical instruments to study potential associations between a large-scale natural disaster and regional air quality. Accordingly, we conducted a spatial and temporal evaluation of BTEX in ambient air in a large area of Eastern North Carolina after Hurricane Florence. This hurricane was associated with a massive rain event causing not only major damage to the environment but also major disruption to anthropogenic activities. Using mobile sampling of BTEX (from a vehicle-mounted analyzer by Entanglement Technologies, San Bruno, CA, USA) and model predictions for ambient air quality parameters (using software by BreezoMeter, Haifa, Israel), we analyzed the concentrations of both air toxics and major criteria air pollutants along major roadways in the impacted area in the immediate aftermath of the hurricane, as well as at four and eight months thereafter. We also examined the spatial relationships between the concentrations of criteria air pollutants and BTEX with potential point sources immediately after Florence and at two four-month intervals after the storm.

## 2. Materials and Methods

### 2.1. Study Area

**Figure 1:**
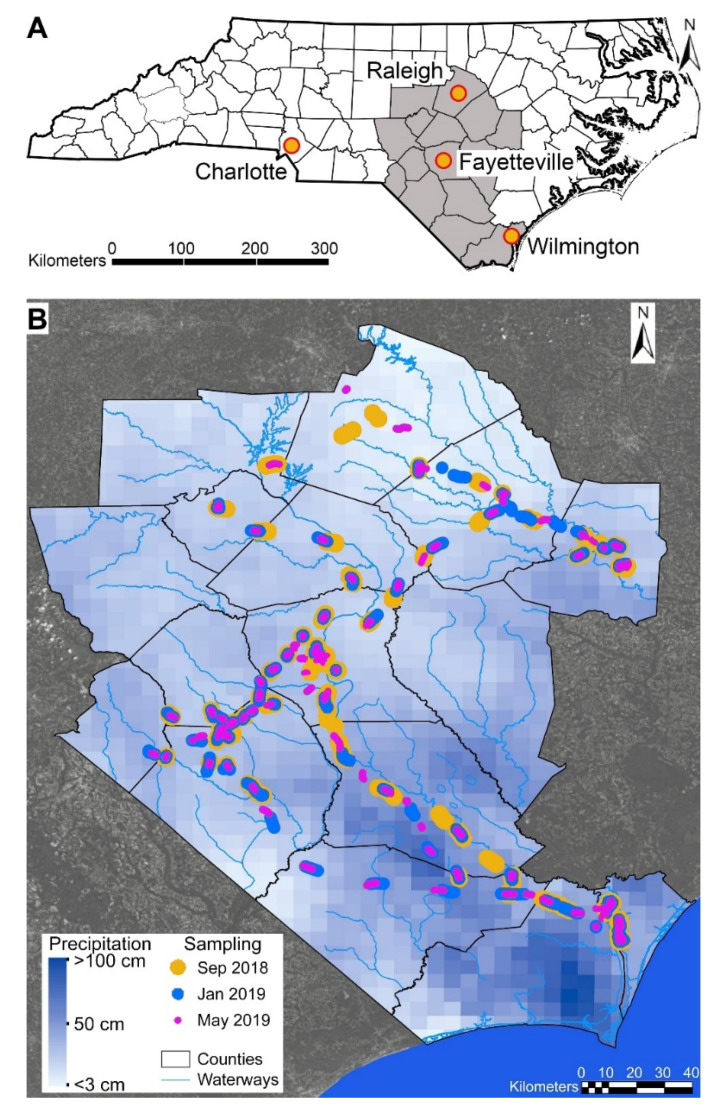
Study area map of North Carolina. (**A**) The map of North Carolina with counties indicated. Shaded in gray are counties where the study was conducted. Major population centers are shown. (**B**) Physical sampling locations for each time period in September 2018 (yellow), January 2019 (blue) and May 2019 (pink) are shown (different widths are so that samples from different dates at the same location can be seen clearly). Overlaid is a precipitation density contour map for the rain totals (see inset for color coding) associated with Hurricane Florence in September 2018. Areas outside of the study area are shaded in gray.

The state of North Carolina (Figure 1A) is located in the southeastern region of the United States of America. North Carolina’s summer temperatures have an average high of 89 °F (31.6 °C) and low of 68 °F (20 °C) and winters have an average high of 53 °F (11.6 °C) and low of 32 °F (0 °C). North Carolina’s weather is dominated by the Bermuda High, where a high-pressure system forms over the Atlantic Ocean during the summer that often leads to many weeks of hot, humid weather and frequent hurricanes in the fall. The average annual precipitation in North Carolina is 45” (1143 mm) and occurs throughout the year. Catastrophic rain events occur primarily in the eastern part of the state and are associated with hurricanes originating in the Atlantic Ocean. For example, Hurricane Florence made landfall in North Carolina on 14 September 2018 as a Category 1 hurricane with sustained winds of 90 mph (150 km/h). Due to the slow movement of Hurricane Florence after landfall, heavy rain fell throughout the Carolinas for several days and widespread flooding was recorded along a long stretch of the North Carolina coast and extended inland over 150 km reaching the cities of Raleigh and Fayetteville. Over about 3 days, Florence-associated rainfall reached a maximum total of 36 inches (913 mm) in Eastern North Carolina; it is regarded as the wettest hurricane to ever impact the state [17]. Most major roads and highways in the area experienced flooding, with large stretches of major inter-state highways remaining impassable for days after the storm.

### 2.2. Mobile Air Quality Sampling for BTEX

Ambient air concentrations of BTEX were sampled using real-time mobile monitoring along several major public highways in Eastern North Carolina, from Raleigh to Wilmington (Figure 1B). A total of three field sampling campaigns were carried out. The first set of samples was collected from 19 September 2018 to 23 September 2018, immediately after the local roads became passable following Hurricane Florence. The second set of samples was collected from 29 January to 1 February 2019, and the third set of samples was collected from 15 May 2019 to 17 May 2019. For easier identification, samples were named 2018 Sep, 2019 Jan, and 2019 May, respectively. All sampling was conducted between 7 a.m. and 9 p.m., and each data point was time-stamped. Sampling time frame was restricted to daylight hours during the initial campaign because floodwaters were still rising and nighttime driving was deemed to be a safety hazard. Subsequent deployments were designed to match the initial deployment to the greatest extent possible.

Real-time BTEX measurements were performed using an AROMA-VOC (Entanglement Technologies, San Bruno, CA, USA) chemical vapor analyzer mounted on a moving vehicle. The analyzer is a combined thermal desorption unit and cavity ringdown spectrometer (CRDS). Controlled volume samples were collected on a Carbopack B fast flow collector with a mass-flow controller and onboard sampling pump. The sorbent was desorbed at 320 °C and transferred to a Carbopack B small bore focuser tube with nitrogen carrier gas. The temperature of the focuser tube was increased (40 °C to 300 °C in 266 s) under 50 sccm nitrogen flow to create a chromatographic-like separation. The resulting elution was analyzed by CRDS. The broadband CRDS cavity (9 cm, wavelength dependent finesse between 400,000 and 100,000) cycled through a predefined list of wavelengths (1623.08 nm, 1641.35 nm, 1647.75 nm, 1664.14 nm, 1669.02 nm) with approximately 0.5 s dwell time per wavelength and a cavity-locked CRDS rate of 1.6 kHz. Compound identification was performed via peak fitting with compound identification performed via spectral match and arrival time identification. During the course of the study, daily bracketing QA/QC measurements were performed. A 30% difference was used for continuing calibration verification (CCV) measurements, as per US EPA method TO-15. In addition, each measurement underwent internal quality assurance checks. The following parameters are validated for each measurement: sampling flow rate, analyzer core flow rate, analyzer core pressure, sorbent temperatures and thermal ramps, laser lock voltage, elution peak arrival time, elution peak spectral match, elution peak goodness of fit. Failures of any of these fit results were automatically flagged and the data were subsequently reviewed manually. If the data were subsequently determined to be valid the results were included in the analysis, otherwise the results were flagged and listed as 0.

The AROMA Analyzer was mounted in the cargo compartment of a Ford Transit Connect van equipped with a 7.2 kWh battery system with engine charging capability. Atmospheric samples were collected through a 12-foot long ¼” Teflon sample line. A brass sintered filter was used at the sample inlet to prevent foreign matter from entering the sampling line. The sampling inlet was mounted to a mast 20 cm above the vehicle roof at the front right of the vehicle. During operation, the line was continually purged at a flow of 1 slpm.

The results of the quality assurance measurements are summarized in Supplementary Table S1. During measurement period 3, ethylbenzene consistently reported an elevated calibration and failed the QA/QC check. The data collected in measurement period 3 was rescaled by the mean deviation (1.24×) of the calibration verification measurements. With this rescaling, all ethylbenzene measurements passed the acceptance criteria. One Xylene CCV failed in the third campaign. A subsequent measurement passed, which was considered acceptable as per our Quality Assurance Surveillance Plan.

The samples were obtained in groups of contiguous measurements along traveled roadways (Figure 1B), and exploratory analyses showed high correlation of pollutant measured concentrations over these short distances. Thus, for the three sets of measurements and some of the analyses detailed in Section 2.5, we grouped the sampled values into 70–95 clusters, with pollutant concentrations and the latitude–longitude averaged for each cluster. This approach effectively reduced the sample size to the number of clusters, but with little loss of statistical efficiency for later regression analyses.

### 2.3. Modeled Air Pollutant Data for Criteria Pollutants

BreezoMeter (Haifa, Israel) carries out world-wide air pollution estimation calculations based on over 49,000 monitoring stations/sensors in over 92 countries each hour. In order to constantly monitor and improve the accuracy, every hour all of the data points are compared retrospectively to the actual measurements. Modeled air pollution concentrations from BreezoMeter for traffic-related pollutants (NO_2_, PM10, and PM2.5) and ambient air pollutants (CO, NO, NO_2_, NOx, O_3_, PM10, PM2.5, SO_2_) in places and at times concurrent with data for the three sampling campaigns mentioned above (2018 Sep, 2019 Jan and 2019 May) were used for comparative analysis.

BreezoMeter considers multiple sources of air quality information to estimate concentrations of each pollutant at a particular location. The layers of information that are currently used include (i) government-operated monitoring stations; (ii) traffic patterns from millions of connected cars (e.g., speed of the cars); (iii) various dispersion models (e.g., the CAMS ensemble model of the ECMWF, which has a global model (0.4 degrees resolution) and a regional EU model (0.1 degrees resolution)); (iv) information on active fires and smoke models (different models at different locations); (v) meteorological information; (vi) land cover; (vii) satellite-derived pollutant measurements; (viii) local low-cost sensor data (where available from government sources). Data are refreshed hourly.

BreezoMeter deploys complex proprietary algorithms and machine-learning techniques to combine all these data sources together and produce real-time air quality information that is available to the public at https://www.breezometer.com/ (accessed on 9 December 2021) For the governmental monitoring stations layer, BreezoMeter first puts the information through a strict quality assurance process to ensure accurate real-time information. Because most governmental stations have an inherent delay in reporting data, BreezoMeter uses proprietary machine-learning algorithms to predict the air quality concentration in real-time. BreezoMeter then conducts an interpolation between all raw data layers and cross validates the predictions with additional models to ensure the most accurate and representative values. The low-cost sensor data available from government channels are integrated indirectly into the model predictions by ‘calibrating’ the data, based on many sensors, in order to ensure that the data refresh time is minimal (hourly). Other models such as those specifically for fires or dust, are integrated as well to ensure a representative report in cases of sudden events with no stations in the vicinity of the event. All layers are combined together, cross-validated and calculated on a grid of 0.004 deg (500 m at the equator). BreezoMeter validates the data using the leave one out cross validation (LOOCV) method: a simple subtraction of our model’s results from each station’s measurement (once the measured data have been reported after several hours’ delay) without the use of that station’s data. The LOOCV method provides an examination of two aspects of BreezoMeter’s algorithm: spatial and temporal. 

### 2.4. Temporal and Geospatial Analysis of the Air Quality Data

Overall temporal differences among the three sampling campaigns (irrespective of location) were assessed using one-way ANOVA followed by Tukey’s multiple comparison tests in Graphpad Prism (Version 9.1.1) and linear trend in R (Version 4.0.5). For visualization, ArcGIS 10.1 software (ESRI, Redlands, CA, USA) was used to conduct spatial analysis of all data. Specifically, for spatial interpolation, kriging within the geostatistical analyst extension tool was used to model the spatial distribution of all chemicals. Individual maps of each measured and modeled chemical were created using ordinary kriging and default parameters determined by the geostatistical wizard. Each raster layer produced from kriging was extracted along the sampling route with a 0.5 km buffer for quantitative comparison of data, but map images utilized a separate 2 km buffer so as to improve visibility. The traveled road network was extracted from road GIS data from the North Carolina state government Department of Transportation. Additionally, to produce better visualization and comparable results, all kriged values were classified into 10 class percentile values from min to max and were extracted with the same boundaries and resolutions.

Measured concentrations of BTEX were compared to US EPA regional screening levels (RSL) for residential ambient air quality, which are health-based screening levels for residential exposure. Specifically, RSL values for residential ambient air were as follows: benzene = 0.36 μg/m^3^ (1 × 10 ^−6^ cancer risk); toluene = 5200 μg/m^3^ (hazard quotient [HQ] = 1); ethylbenzene = 1.1 μg/m^3^ (1 × 10 ^−6^ cancer risk); xylenes = 100 μg/m^3^ (HQ = 1). Criteria air pollutant concentrations were compared to U.S. EPA National ambient air quality Standards (NAAQS). Specifically, the lowest NAAQS criteria were used for comparison: PM2.5 = 12 μg/m^3^ (primary, 1 year); PM10 = 150 μg/m^3^ (primary and secondary 24 h); NO_2_= 53 ppb (primary and secondary 1 year); SO_2_ = 75 ppb (primary 1 h); O_3_ = 70 ppb (primary and secondary 8 h); CO = 9000 ppb (primary 8 h).

All original data are available in an Excel spreadsheet in the Supplementary Materials.

### 2.5. Clustering and Spatial Correlation with Fueling Station Locations

Fueling station locations were obtained from North Carolina Multi Hazard Threat Database and North Carolina Department of Environmental Quality Online GIS [18].

The mobile pollutant sampling (see Section 2.2) was conducted in short discrete time intervals (minutes), and for each such interval a single GPS coordinate was used as a sampling point location. The number of sampled points were 125, 101, and 109 for September 2018, January 2019 and May 2019, respectively. Upon close examination of the data, some of the pairs of sampling locations (contiguous in time) were so close together spatially that we reasoned they could be joined into a single sampling point. To automate this process and prevent bias, we used single-linkage hierarchical clustering of the spatial point locations using *hclust* in R and Euclidean spatial distance, which joined a minority of the paired nearby sampling points, implemented using the R *cuttree* command. Although there was some subjectivity in declaring the number of final sampled points, the number of clusters was chosen separately from any downstream analysis. After the joining process there were 90, 70, and 85 sampling points for September 2018, January 2019 and May 2019, respectively. These “clustered” data are available as a separate Excel spreadsheet in the Supplementary Materials.

Our spatial analysis approach was motivated by hypothesis testing for relation of pollutant concentrations to fueling stations as potential point sources rather than developing detailed physical models to determine how concentration of a pollutant decays with distance from the point source. However, three-dimensional modeling to construct such models would require airflow measurements that were beyond the scope of this study. We used spatial regression modeling for the combined effects of fueling station point sources, which requires a scale for the decay of effects with distance. For parsimony, we applied a simple inverse distance power law to represent the impact of pollutant point sources, recognizing that a great number of factors contribute to diffusion in a pollutant-specific manner [19]. Pollutant concentrations were summed over the contributions from overlapping point sources. Thus for each cluster *i*, with $d_{ig}$ denoting distance from cluster location *i* to fueling station *g*, a predictor value $x_{i}=\overset{G}{\underset{g=1}{\sum }}1/d_{ig}^{f}$ was computed for power exponent *f*, summing over all point sources (fueling stations) *g* = 1,…,*G*.

As an initial data exploration step, for each dataset we computed simple Spearman rank correlations between each pollutant concentration *y* and the fueling station predictor *x*, varying the power *f* across a range 0.5–6.0 (see Figure S1). We reasoned that a fraction of pollutants would show significant effects, and for each *f* we computed the 80th percentile of the correlations across pollutants for each dataset. We note that the rank correlations are invariant to monotone transformations of *y*, but not to the power value *f* because summation is performed prior to ranking. Each of the three datasets showed a peak correlation for *f*, and these values ranged from 1.2–2.4. The correlation quantile did not vary dramatically in this range, and for large values of *f* appeared to stabilize (data not shown). The fact that the correlations drop only modestly for large *f* likely reflects that the concentration drops very quickly for many of the pollutants [19]. Based on our results, for simplicity we used *f* = 2.0 throughout the analysis, corresponding to an inverse distance squared weighting. However, we note that the validity (false positive rate) of hypothesis testing does not depend on the choice of *f*.

To perform formal hypothesis testing of the locations of fueling stations vs. pollutant measured concentrations, we employed linear spatial regression for pollutant concentrations vs. our summed inverse distance measure to surrounding fueling stations. Essentially, this approach involves a linear regression for pollutant concentrations vs. the fuel station distance measure. However, spatial autocorrelation (the tendency for pollutant concentrations to be similar in nearby locations) might be present for reasons unrelated to the fueling station distance measure, which could increase false positive findings unless handled appropriately. Spatial regression via the function *lagsarlm*, as implemented in the R *spatialreg* package [20], can estimate the underlying fueling station distance vs. pollutant concentration regression relationship and obtain appropriate standard errors while accounting for the autocorrelation. The approach uses maximum likelihood and a spatially lagged model to account for autocorrelation [21], so that the final regression *p*-value is appropriately reflective of the relationship between pollutant concentration and the fuel station distance measure.

Initial estimation of spatial correlation in pollutant concentrations under a null model (i.e., without considering the fuel station point sources) indicated clear spatial correlation for many pollutants, even after the cluster reduction, and so we employed *k*-nearest neighbor weights as the *listw* argument for the spatial autocorrelation used in *lagsarlm*. *p*-values for the fueling station predictor and for residual autocorrelation (computed in the model using maximum likelihood approach) were obtained as *z*-statistics for each dataset and pollutant with an accompanying two-sided *p*-value. We focus on *p*-values instead of the SARLM coefficients (which represent the effect of summed inverse power weighted coefficients on pollutant concentrations) due to the inherent lack of interpretability of the predictor in terms of units (although the fuel station locations may be important) and the fact that we are primarily performing hypothesis testing.

For each dataset, we increased *k* sequentially in the model including the fueling station predictor until no significant autocorrelation effects remained. As multiple pollutants were examined, some pollutants could exhibit apparently significant autocorrelation *p*-values for remaining autocorrelation simply by chance. Thus, in each dataset we first corrected for multiple comparisons by computed the false discovery *q*-value using the *p.adjust* function in R with the Benjamini-Hochberg adjustment [22]. For each dataset we increased *k* sequentially until no pollutant exhibited autocorrelation *q* < 0.05. For the September 2018, January 2019 and May 2019 datasets, the respective number of neighbors that achieved this criterion was *k* = 2, 5, and 2, respectively. In order to be conservative in our correction for autocorrelation, for the final analysis we used the maximal value *k* = 5 throughout. The approach is likely conservative, as the most auto-correlated pollutant essentially determines the autocorrelation neighborhood used. As an additional check for residual autocorrelation, we implemented Moran’s I method [23] using *moran.test* in the R *spdep* package on the residuals using a 1/distance^2^ weighting function under 10,000 random permutations, with no pollutant showing significant residual autocorrelation.

To identify the pollutants with the most consistent correlation with the fueling station point sources, for each pollutant we computed summed *z*-statistics across the three datasets for the fueling station predictor,

(1) $$z_{total}=\left(z_{Sep2018}+z_{Jan2019}+z_{May2019}\right)/\sqrt{3},$$

with corresponding *p_total_* and *q_total_* (*q*-values from the set of *p_total_*).

## 3. Results

### 3.1. Spatial and Temporal Trends in BTEX after Hurricane Florence

**Figure 2:**
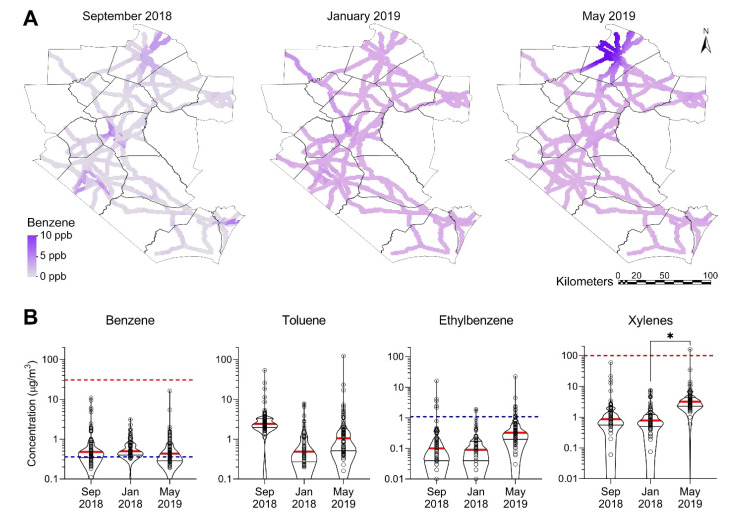
Spatiotemporal distribution and concentrations of BTEX along the major roadways in the study area. (**A**) Kriged maps showing spatial distribution of benzene for three sampling campaigns. Color gradients (see inset) show concentrations. See Supplemental Figures S2–S4 for the distribution of other VOCs. (**B**) Violin plots showing temporal distribution of BTEX concentrations for three sampling campaigns (red thick line = median; thin black lines = quartiles, dots = individual sample data) in comparison to screening levels (red dashed line = non-cancer; blue dashed line = cancer) for each pollutant. If no line is shown on a chart, the screening value has not been established or the screening value is greater than the range of data points shown. The asterisk (*) denotes statistically significantly different (*p* < 0.05) by one-way ANOVA followed by Tukey’s multiple comparison tests.

This study focused on spatial and temporal changes in regional air quality both immediately after Hurricane Florence and up to eight months afterwards. We collected measurements of air toxics in the form of BTEX species over both space (eastern and central North Carolina) and time. Overall summaries of measured BTEX concentrations for all three time points (2018 September, 2019 January and 2019 May) are presented in Table 1 and visualized in Figure 2. The highest mean concentrations were found in the first sampling period (2018 September) for benzene and toluene and the third sampling period (2019 May) for ethylbenzene and xylenes.

When compared to the US EPA RSLs, we found that measured benzene levels exceeded RSL in 62 to 87%, and for ethylbenzene in 3 to 12% of the sampling locations. No sampling location exceeded the RSL for toluene and only 1% (2019 May) for xylenes. The benzene and ethylbenzene RSLs are based on screening benchmarks of 10^−6^ cancer risk for a lifetime exposure in a residential exposure scenario; all the measured levels would be below the less stringent benchmark of 10^−4^ cancer risk.

Because benzene was the pollutant that exceeded the RSL most frequently, we show the spatial distribution of benzene levels across the study area (Figure 2A) at three sampling time intervals (2018 September, 2019 January and 2019 May); similar data for other species are shown in Supplemental Figures S2–S4. High benzene levels appear to be localized in urban centers in the first sampling period immediately after the hurricane, with much lower levels in other areas. At the same time, benzene appeared to be more evenly distributed along the traveled roadways in the latter sampling periods (except in the northern part of the region). However, comparisons of spatial distributions should be treated with caution because not every roadway was sampled in every sampling campaign, and Kriging assumes a continuous distribution over the entire area.

The violin plots in Figure 2B show the distribution of the BTEX data and its probability density. All pollutants had a very wide range in measured values with a few points that were an order of magnitude higher than the median. At the same time, many more sampling points were far lower than the median levels. Statistically significant differences between sampling time points were only found for xylenes (including a positive trend *p* = 0.03 from September 2018 to May 2019).

### 3.2. Spatial and Temporal Trends in Criteria Air Pollutants after Hurricane Florence

**Figure 3:**
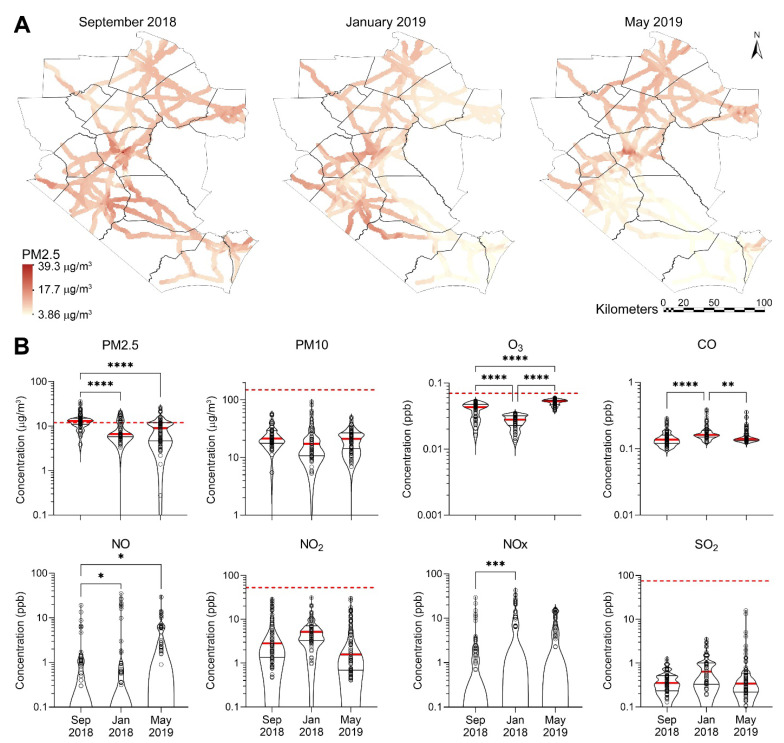
Spatiotemporal distribution and concentrations of criteria air pollutants along the major roadways in the study area. (**A**) Kriged maps showing spatial distribution on PM2.5. Color gradients (see inset) show concentrations. See Supplemental Figures S5–S14 for the distribution of other criteria air pollutants. (**B**) Violin plots showing temporal distribution of BTEX concentrations for three sampling campaigns (red thick line = median; thin black lines = quartiles, dots = individual sample data) in comparison to screening levels (red dashed line = non-cancer) for each pollutant. If no line is shown on a chart, the screening value has not been established or the screening value is greater than the range of points shown. Asterisks denote statistically significant differences by one-way ANOVA followed by Tukey’s multiple comparison tests (* = *p* < 0.05, ** = *p* < 0.01, *** = *p* < 0.001, **** = *p* < 0.0001).

For further comparison and corroboration of the patterns in BTEX data, we extracted modeled estimates of criteria air pollutants (both overall and traffic-related), at the same locations and times as our BTEX measurements. An overall summary of these modeled concentrations for all 3 time points (September 2018, January 2019 and May 2019) is presented in Table 2 and visualized in Figure 3. As with BTEX, the temporal patterns differed across the 8 pollutants, with mean concentrations highest in the first sampling period for PM2.5 and PM10, the second sampling period for CO, NO_2_, and NO_X_, and the 3rd sampling period for the remaining pollutants O_3_ and SO_2_.

We found that PM2.5 levels exceeded the most stringent NAAQS (one-year primary criteria of 12 μg/m^3^) at between 22% and 66% of the sampling locations. No sampling location exceeded the NAAQS for any of the other criteria pollutants. Interestingly, while the PM2.5 concentrations at the 2nd and 3rd sampling times were in line with annual averages of 7–10 μg/m^3^ in urban areas of North Carolina, even when accounting for seasonal differences [24], the values immediately after Hurricane Florence were somewhat higher.

Because PM2.5 was the only pollutant that exceeded the NAAQS, we show the spatial distribution of PM2.5 levels across the study area (Figure 3A) at three sampling time intervals (September 2018, January 2019 and May 2019), and similar data for other criteria air pollutants are shown in Supplemental Figures S5–S14. When comparing these three time periods, the concentration of PM2.5 appears to decline with time, particularly in the south-eastern region of the sampling area. A degree of concentration in urban areas is also evident, but the amounts do not become more spatially homogeneous over time as much as for benzene. The patterns across the other criteria air pollutants, however, differed from each other (see Supplemental Figures S5–S14). However, as with BTEX, comparisons of spatial distributions should be treated with caution due to relatively sparse sampling and the assumptions underlying Kriging-based interpolation.

The violin plots in Figure 3B show the distribution of the criteria pollutant data and their probability density. PM2.5, PM10, O_3_, and CO tended to have narrower ranges of concentrations. Different temporal patterns were also evident. For instance, while PM 2.5 trends downward (*p* < 0.0001), the trend is either absent (PM10, NO_2_) or reversed (O_3_, NO, NO_x_, SO_2_; *p*-values are ranging from <0.0001 to 0.03) for all other chemicals.

Correlations between BTEX compounds and modeled ambient and traffic criteria air pollutants’ concentrations are shown in Supplemental Figures S15 and S16. There was a positive correlation among BTEX compounds and among NO_2_, PM10 and PM25, which is consistent with a common emission source for these compounds, such as traffic [25,26].

### 3.3. Spatial Correlation with Fueling Stations

**Figure 4:**
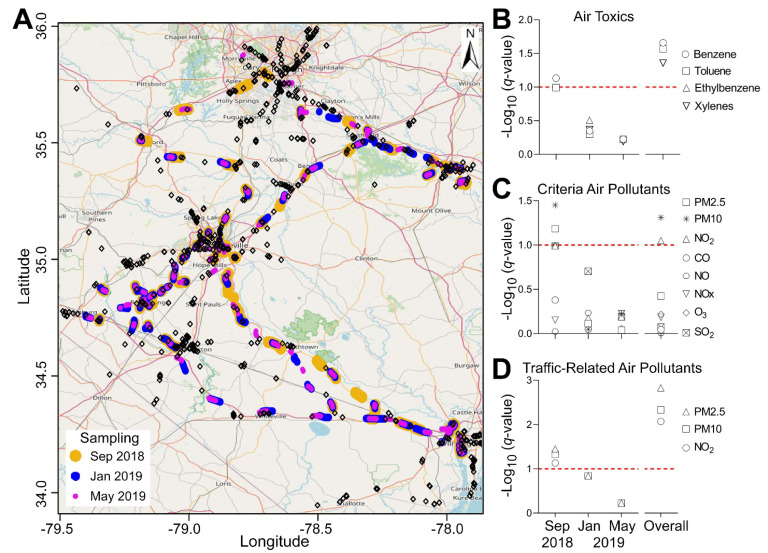
Spatial association between each concentration of each pollutant evaluated in this study and fueling stations at each of the sampling periods. (**A**) Map showing sampling points for all three time periods (see color map as an inset) in relation to the fueling station locations (black diamonds). (**B**–**D**) Significance of the spatial association (*q*-values) between air pollutants and fueling stations. Horizontal dotted line shows false discovery rate of 10%. (**B**) BTEX, (**C**) criteria air pollutants, and (**D**) traffic-related criteria air pollutants.

The spatial distribution of fueling stations and BTEX sampling locations is shown in Figure 4A, with false discovery rate-corrected significance expressed as *q*-values [22] for the fueling station site association with various measured air pollutants shown in Figure 4B–D. Immediately after Hurricane Florence (September 2018), there tended to be a greater association between gas stations near the hotspot locations (higher concentration areas) as compared to lower concentration areas. This correlation tended to be stronger when considering total values across the three datasets, with 9 pollutants having *q* <0.1. Interestingly, both benzene and PM2.5, which had greater concentrations and apparent clustering immediately after the hurricane (September 2018), also exhibited greater associations with fueling stations.

## 4. Discussion

Our primary objective was examining the spatial and temporal patterns of air toxics (e.g., BTEX) and criteria air pollutants in relation to widespread flooding in the aftermath of Hurricane Florence. Based on either the mean or median concentrations of the individual BTEX species, we found the following rank order of these toxics: X~T > B > E. Due to the more stable nature of toluene and benzene as compared to xylene and ethylbenzene, we had expected a ranking order that is similar to other studies where they reported the highest concentration of toluene, followed by m/*p*-xylene, benzene, o-xylene, and ethylbenzene [27,28,29]. Based on the analytical method used for conducting this mobile air quality sampling, we reported total xylene concentration rather than levels for m/*p*-xylene and o-xylene separately; this may be the primary reason we found xylenes to be as high as toluene.

**Figure 5:**
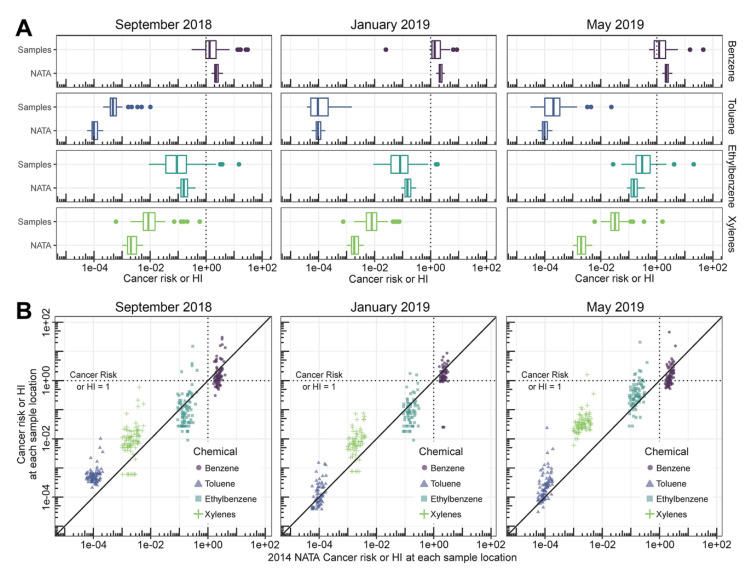
Comparison of cancer risk (per million) or hazard index (HI) based on our sampling data and those estimated from the 2014 National Air Toxics Assessment (NATA) (U.S. EPA 2014). Cancer risk and HI for our sample locations were calculated as the ratio between the sample concentration and the corresponding RSL (see Figure 1 legend). For NATA-based risks, sample locations were then mapped to corresponding census tracts, and NATA cancer risk (for benzene [circle] and ethylbenzene [square]) and HI (for toluene [triangle] and xylenes [cross]) for that census tract extracted. (**A**) Boxplot comparison. (**B**) Scatter plot comparisons. The dotted lines show the benchmark of cancer risk of 10^−6^ and HI = 1; the diagonal line is equality.

When exposures were considered in the context of human health risk, we found that B > E > X > T, showing the importance of comparing concentrations to health benchmarks when interpreting air monitoring data. These results are broadly consistent with air toxics emissions data and modeling performed under EPA’s National Air Toxics Assessment (NATA), which use the same toxicity values but with more complex exposure scenarios as compared to RSLs [30]. As shown in Figure 5, the latest available NATA estimates of cancer risk and hazard indices at the sample sampling locations shows the same ordering of risks (B > E > X > T). Additionally, benzene shows NATA-estimated risks above the relevant health-based benchmark (HI > 1 or cancer risk per million > 1), consistent with our results (Table 1). Although median values for xylenes were similar, we found a small percentage of samples with concentrations of xylenes above the benchmark, while NATA showed all values below 1. This is not unexpected since NATA are based on annual averages rather than spot samples.

With respect to spatial distribution, in general, benzene appeared to be more highly clustered in urban areas immediately after hurricane Florence as compared to January 2019. This tendency persists in May 2019 with the main exception that benzene concentrations at this time point appear to be far higher in the northern part of the sampling area, in the metropolitan area around Raleigh, NC. We note several possible reasons for this divergence. First, visual observations by the mobile laboratory operators noted very sparse traffic during the first deployment immediately following the hurricane (September 2018). In addition, the vehicle distribution being atypical with a large number of military vehicles as compared to the predominance of light-duty vehicles that would be expected in this area. Traffic patterns and vehicle type distribution reverted to more typical patterns upon return sampling trips, with more dense traffic around Raleigh, NC. In addition, we note that due to some flooding-related road closures in the first sampling period, the exact sampling paths differed somewhat among the three time periods; therefore, precise comparative analysis of spatial and temporal patterns along the edges of the sampling area cannot be interpreted with confidence. Therefore, the apparent “hot spot” in the northern-most edge of the study area may have been influenced by the greater extent of sampling in that direction in May 2019 (i.e., this was the only sampling campaign that extended that far north). This observation suggests that the results of kriging and other interpolation methods should be interpreted with caution when applied to mobile sampling due to inherent differences in sampling locations over time.

The clustering of higher concentrations of benzene was corroborated by their association with inverse distance from fueling stations. While this clustering may be simply due to greater numbers of fueling stations being a proxy for more urban areas, is it also plausible that benzene might actually be directly emanating from fueling stations. Specifically, as vent pipe emissions from storage tanks have been recently implicated in short-term exceedances of benzene exposure limits, and emissions would be expected to increase when tanks are not being used, as would have been expected during the immediate aftermath of the Hurricane [31,32,33]. For both benzene and PM2.5, it is also possible that the greater association with gas station locations immediately after Florence was due to traffic being more clustered towards urban centers during that period when many roads were closed and much of the population was engaged in recovery operations. Additionally, for PM2.5, the heavier-than-usual presence of diesel-powered military vehicles assisting in recovery operations may have also contributed to their concentration near urban areas. The greater concentration of traffic and higher proportions of diesel vehicles would also explain our observation that PM2.5 concentrations exceeded regulatory limits much more often immediately after Florence as compared to other time periods.

This study had a number of important limitations. First, a major challenge for most disaster research response (DR2) studies is that there is little historical information available for comparisons to the newly collected data. It is typical that natural disasters are associated with community concerns about potential chemical contamination as the result of flooding of chemical manufacturing or storage sites, this was also the case for hurricane Florence-associated flooding in eastern North Carolina. Even though most such concerns associated with hurricane Florence were focused on potential oral route exposures from spills of coal ash and animal waste into the local watersheds [34,35], air quality concerns were equally prevalent. Because of the sparsity of the fixed-site air quality monitoring networks and their emphasis on ambient air quality metrics, it is difficult to compare the new measurements in this study across time and space. However, to address this limitation, we collected time-trends through repeated sampling campaigns over a period of up to 8 months. Even though there were inconsistencies in some sampling areas due to post-hurricane road closures, the attempt to establish a typical profile of air quality in the same large area allowed us to draw conclusions about hurricane Florence-related component in these trends. The post-event “background” is frequently the only option for determining the effects of the disaster as compared to pre-existing contamination [35,36,37,38]. In addition, studies that provide a temporal analysis of environmental exposures [36,37] establish the point of reference for the future investigation and will enable more expedient determination of the potential impacts.

Second, studies of air quality at the scale of tens to hundreds of kilometers, similar to our study that covered hundreds of kilometers of the roadways in a large geographical area, are also a challenge. Air pollution is typically studied at the macro-scale, where satellite imaging and the networks of fixed monitors result in the regional exposure estimates [39], or at the micro-scale, where personal monitors provide high-resolution data for a particular person or locale [40]. Recent studies of high-resolution mobile monitoring of air pollutants compared to the data from a dense fixed-site network showed that a combination of the two can effectively fill in the gaps in spatial patterns and that the instrument noise becomes the limiting factor to increasing the precision of the estimates [41]. Indeed, mobile monitoring of various air pollutants has become a promising technology [42,43], and these measurements can inform studies of associations between air pollution and human health [44]. However, most such studies focused on major criteria air pollutants and the data on VOCs is far less developed, with substantial heterogeneity even due to “normal” spatial and temporal variability [45,46]. Thus, even though our study may not be as precise in estimating the fine-scale pollutant estimates and is limited to passable roadways that may not necessarily be representative of population-wide exposures, it does provide a valuable point of reference for the overall potential hot-spots and ranges in concentrations detectable over time and space. While we did use kriging to interpolate across spatial scales, more sophisticated techniques such as land use regression modeling may be useful for predicting VOC hotspot locations [47].

## 5. Conclusions

In summary, this study investigated the spatial and temporal variation of BTEX and criteria air pollutants in a large geographical area in eastern North Carolina to examine the potential impacts of wide-spread flooding associated with Hurricane Florence. Evidence of the air quality effects of Hurricane Florence was strongest for benzene and PM2.5. For both benzene and PM2.5, there appeared to be an association between higher concentrations and fueling stations and, by proxy, urban centers. Additionally, concentrations of both benzene and PM2.5 exceeded ambient air criteria routinely, with exceedances of benzene occurring in all sampling periods and those of PM2.5 mostly occurring immediately after Florence. Overall, our study suggests that benzene and PM2.5 in particular may be affected by flood-related disasters and deserve particular attention in future DR2 studies due to their potential human health impact.

## Figures and Tables

**Table 1:** Summary statistics of BTEX concentrations.

	Min.	1st Qu.	Median	Mean	3rd Qu.	Max.	%>RSL ^a^
**Benzene (μg/m^3^)**							
September 2018	0.11	0.35	0.48	1.0	0.82	11	74%
January 2019	0.0091	0.40	0.50	0.69	0.80	3.1	87%
May 2019	0.19	0.29	0.44	0.81	0.74	16	62%
**Toluene (μg/m^3^)**							
September 2018	0.0061	2.0	2.4	3.7	3.2	53	0%
January 2019	0.0030	0.28	0.49	1.0	1.1	7.9	0%
May 2019	0.16	0.52	1.1	3.3	1.8	120	0%
**Ethylbenzene (μg/m^3^)**							
September 2018	0.010	0.04	0.1	0.46	0.22	16	6%
January 2019	0.010	0.042	0.09	0.18	0.17	1.9	3%
May 2019	0.030	0.20	0.33	0.80	0.64	23	12%
**Xylenes (μg/m^3^)**							
September 2018	0.061	0.58	0.87	2.3	1.5	59	0%
January 2019	0.076	0.51	0.79	1.3	1.2	7.6	0%
May 2019	0.60	2.3	3.2	5.9	4.4	160	1%

^a^, Regional Screening Levels (RSL) for residential ambient air were as follows: benzene = 0.36 μg/m^3^ (1 × 10 ^−6^ cancer risk); toluene = 5200 μg/m^3^ (hazard quotient [HQ] = 1); ethylbenzene = 1.1 μg/m^3^ (1 × 10 ^−6^ cancer risk); xylenes = 100 μg/m^3^ (HQ = 1).

**Table 2:** Summary statistics of predicted concentrations for criteria air pollutants.

	Min.	1st Qu.	Median	Mean	3rd Qu.	Max.	%>NAAQS ^a^
**PM2.5 (μg/m^3^)**							
September 2018	3.8	11	13	14	15	36	66%
January 2019	0.00	5.7	6.7	9.2	13	21	30%
May 2019	0.28	4.7	9.0	9.2	12	26	22%
**PM10 (μg/m^3^)**							
September 2018	5.5	18	21	24	29	58	0%
January 2019	0.00	11	17	22	26	93	0%
May 2019	7.0	14	21	22	26	50	0%
**O_3_ (ppb)**							
September 2018	16	31	43	40	48	54	0%
January 2019	0.00	23	28	26	32	35	0%
May 2019	38	47	53	52	55	60	0%
**CO (ppb)**							
September 2018	99	121	137	144	160	286	0%
January 2019	0.00	150	162	178	187	387	0%
May 2019	127	133	141	158	169	355	0%
**NO (ppb)**							
September 2018	0.00	0.00	0.00	1.06	0.96	19	NA
January 2019	0.00	0.00	0.00	3.3	1.0	35	NA
May 2019	0.00	0.00	0.00	3.1	5.9	29	NA
**NO_2_ (ppb)**							
September 2018	0.47	1.4	2.8	5.9	7.5	29	0%
January 2019	0.00	3.3	5.2	6.2	7.1	31	0%
May 2019	0.41	0.69	1.6	4.5	6.1	30	0%
**NO_X_ (ppb)**							
September 2018	0.00	0.00	0.00	2.0	1.9	29	NA
January 2019	0.00	0.00	0.00	6.4	10	43	NA
May 2019	0.00	0.00	0.00	4.2	7.4	16	NA
**SO_2_ (ppb)**							
September 2018	0.00	0.23	0.35	0.42	0.52	1.3	0%
January 2019	0.00	0.32	0.61	0.81	0.99	3.51	0%
May 2019	0.00	0.22	0.34	0.89	0.59	16	0%

^a^, National ambient air quality Standards (NAAQS) used for these comparisons: PM2.5 = 12 μg/m^3^ (primary, 1 year); PM10 = 150 μg/m^3^ (primary and secondary 24 h); NO_2_: 53 ppb (primary and secondary 1 year); SO_2_ = 75 ppb (primary 1 h); O_3_ = 70 ppb (primary and secondary 8 h); CO = 9000 ppb (primary 8 h). NA denotes no standards are available.

## Data Availability

Original data as well as clustered data are provided separately as Excel spreadsheets in the Supplementary Materials.

## Supplementary Materials

The following are available online at https://www.mdpi.com/article/10.3390/ijerph19031757/s1. Original data Excel spreadsheet: Supplementary data_original.xlsx. Clustered data Excel spreadsheet: Supplementary data_clusteranalysis.xlsx. Table S1. QA/QC Summary of Mobile Air Quality Sampling. Figure S1. For each monthly dataset, pollutant and power law value *f*, the rank correlation *r* was computed between the pollutant concentration and the summed inverse distance values from fuel stations, as described in the text. To account for the possibility that only a fraction of pollutants reflects true association, the 0.8 quantile of the *r* values across pollutants, within each dataset, was used as an index to demonstrate the varying strength of association over different values of *f*. Across the power law values *f*, a global maximum (or local maximum, for Jan 2019) was achieved in the range 1.2–2.4 (with different peak locations for the three monthly datasets). For parsimony, we considered the average of the three curves across the three monthly datasets, which showed a peak close to *f* = 2, and this value was then carried forward in later analysis for all datasets and pollutants. Figure S2. Spatial distribution of Toluene for September 2018, January 2019 and May 2019. Figure S3. Spatial distribution of Ethylbenzene for September 2018, January 2019 and May 2019. Figure S4. Spatial distribution of Xylene for September 2018, January 2019 and May 2019. Figure S5. Spatial distribution of carbon monoxide (CO) for September 2018, January 2019 and May 2019. Figure S6. Spatial distribution of nitrogen monoxide (NO) for September 2018, January 2019 and May 2019. Figure S7. Spatial distribution of nitrogen dioxide (NO_2_) for September 2018, January 2019 and May 2019. Figure S8. Spatial distribution of nitrogen oxides (NO_x_) for September 2018, January 2019 and May 2019. Figure S9. Spatial distribution of ozone (O_3_) for September 2018, January 2019 and May 2019. Figure S10. Spatial distribution of PM10 for September 2018, January 2019 and May 2019. Figure S11. Spatial distribution of sulfur dioxide (SO_2_) for September 2018, January 2019 and May 2019. Figure S12. Spatial distribution of traffic-related nitrogen dioxide for September 2018, January 2019 and May 2019. Figure S13. Spatial distribution of traffic-related PM10 for September 2018, January 2019 and May 2019. Figure S14. Spatial distribution of traffic-related PM2.5 for September 2018, January 2019 and May 2019. Figure S15. The scatter-matrix, and correlation matrix for measured BTEX with modeled NO_2_, PM10, PM25, NO, O_3_, NO_x_, CO, SO_2_. Figure S16. The scatter-matrix, and correlation matrix for measured BTEX with modeled traffic-related NO2, PM10 and PM2.5.
