# Supplementary material for: Spatial and Temporal Analysis of Impacts of Hurricane Florence on Criteria Air Pollutants and Air Toxics in Eastern North Carolina
Source: Int J Environ Res Public Health. 2022 Feb 3;19(3):1757. doi: 10.3390/ijerph19031757 (PMC8835244; doi:10.3390/ijerph19031757)
Supplement: Supplementary file 1 [file ijerph-19-01757-s001.zip › Supplementary Revised/Bhandari et al - Florence-Supplementary-R1.pdf]

Spatial and temporal analysis of impacts of Hurricane Florence on criteria air pollutants and air toxics in Eastern North Carolina

Supplemental Materials

Sharmila Bhandari[1,2], Gaston Casillas[2], Noor A. Aly[1,2], Rui Zhu[3], Galen Newman[3], Fred A. Wright[4], Anthony Miller[5], Gabriela Adler[6], Ivan Rusyn[1,2], Weihsueh A. Chiu[1,2,*]

[1]Department of Veterinary Integrative Biosciences, [2]Interdisciplinary Faculty of Toxicology, and [3]Department of Landscape Architecture and Urban Planning, Texas A&M University, College Station, Texas, 77845, USA

[4]Bioinformatics Research Center, and Departments of Statistics and Biological Sciences , North Carolina State University, Raleigh, North Carolina, 27695, USA

[5]Entanglement Technologies San Bruno CA 94066, USA

[6]Breezometer, Haifa, 3303124 Israel

1. Supplementary data

Original data as well as clustered data are provided separately as Excel spreadsheets.

Original data: Supplementary data_original.xlsx

- These consist of the original mobile sampling data as collected during each sampling campaign, as described in Methods Section 2.2.

Clustered data: Supplementary data_clusteranalysis.xlsx

- These consist of both the mobile sampling data, clustered by location/timepoint as described in Methods Section 2.5, as well as the corresponding modeled criteria air pollution predictions at the corresponding location/timepoint (Methods Section 2.3).

2. Supplementary Tables

Table S1. QA/QC Summary of Mobile Air Quality Sampling

	September 2018	January 2019	May 2019
Total Analysis Runs	133	103	123
CCV Measurements	6	5	6
QA/QC Automatically Flagged Fit results	22	58	8
Cleared in post-analysis	22	27	4
CCV Failures (initial)	0	0	4
CCV Failures After Re-calibration	0	0	1

3. Supplementary Figures

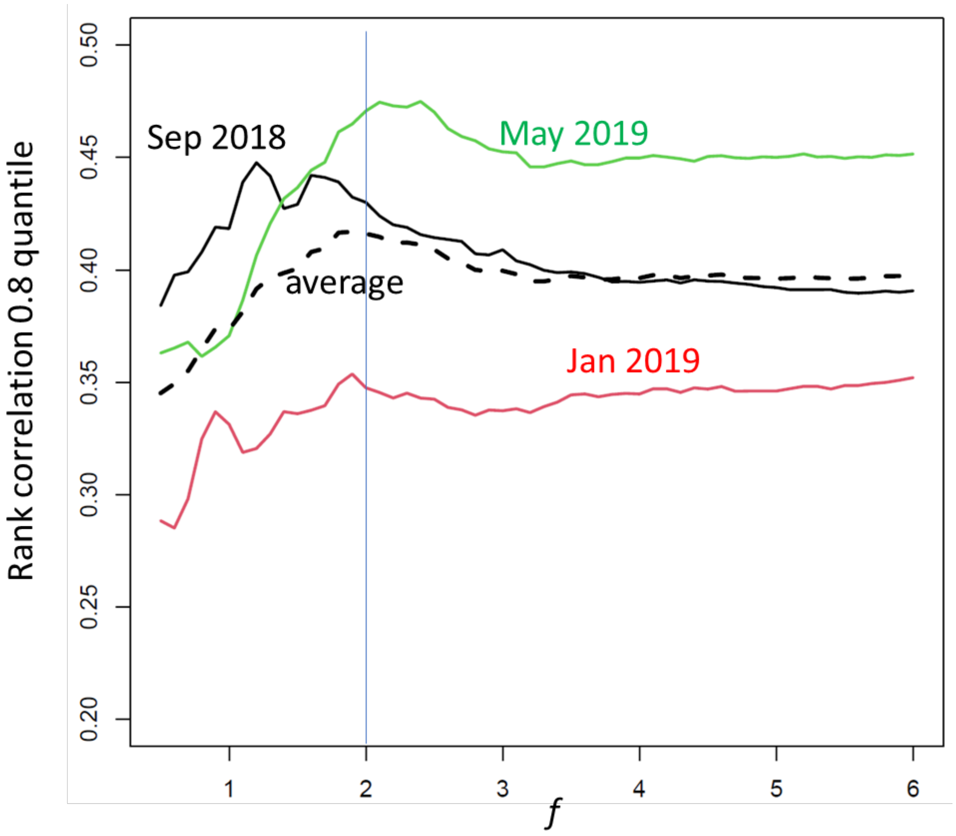

Figure S1. For each monthly dataset, pollutant and power law value *f*, the rank correlation *r* was computed between the pollutant concentration and the summed inverse distance values from fuel stations, as described in the text. To account for the possibility that only a fraction of pollutants reflects true association, the 0.8 quantile of the *r* values across pollutants, within each dataset, was used as an index to demonstrate the varying strength of association over different values of *f*. Across the power law values *f*, a global maximum (or local maximum, for Jan 2019) was achieved in the range 1.2-2.4 (with different peak locations for the three monthly datasets). For parsimony, we considered the average of the three curves across the three monthly datasets, which showed a peak close to *f*=2, and this value was then carried forward in later analysis for all datasets and pollutants.

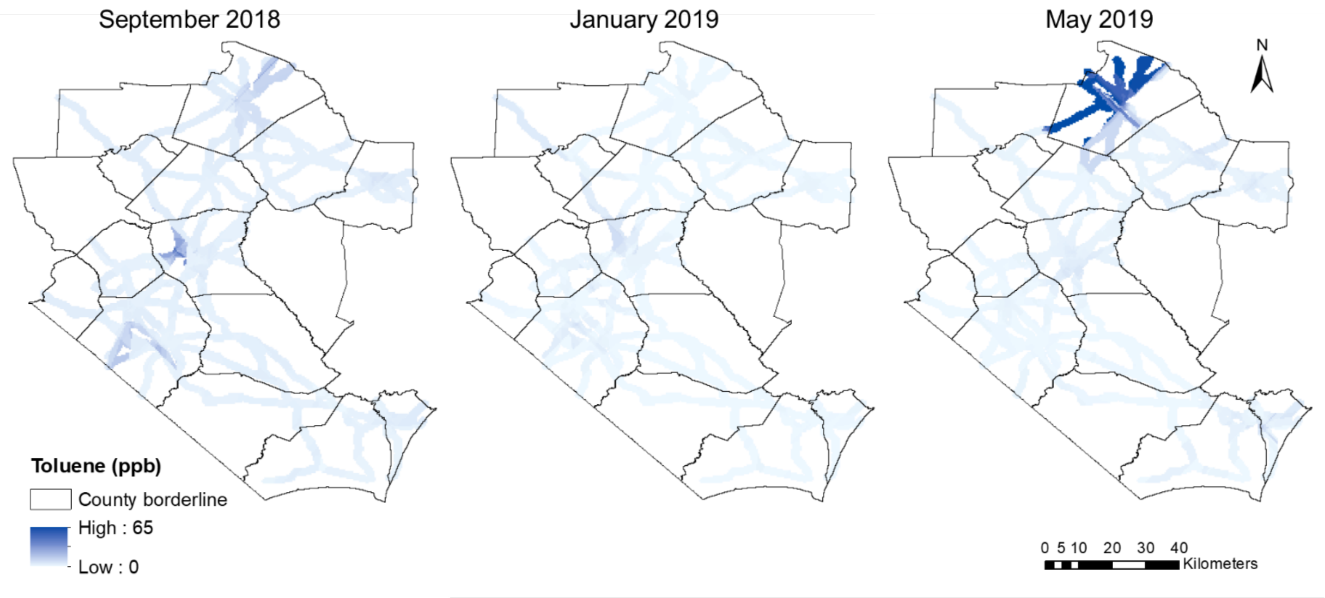

Figure S2. Spatial distribution of Toluene for September 2018, January 2019 and May 2019.

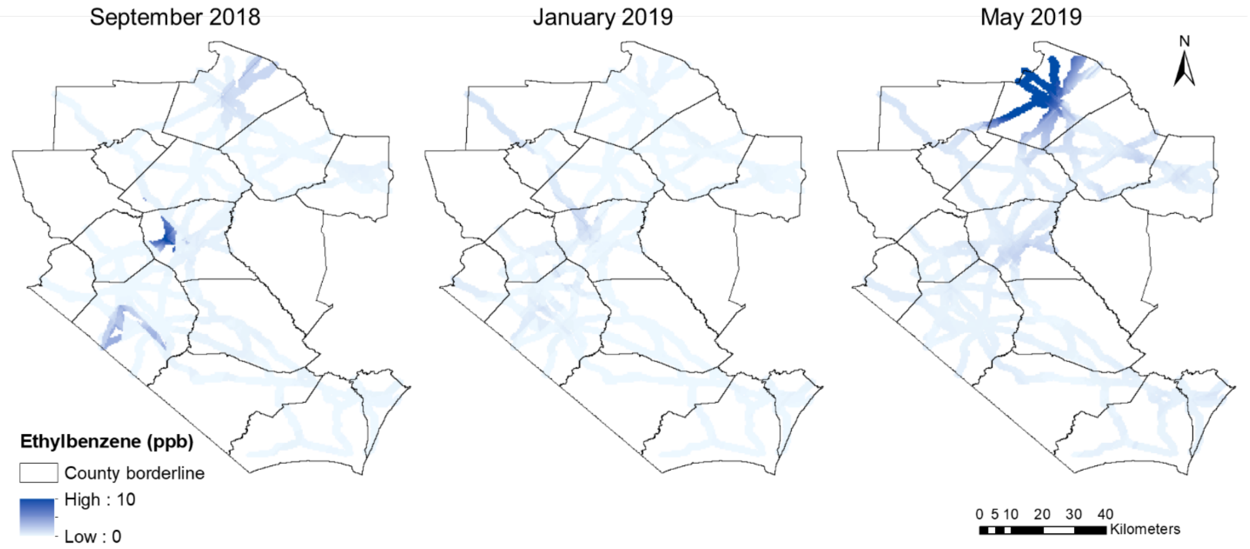

Figure S3. Spatial distribution of Ethylbenzene for September 2018, January 2019 and May 2019.

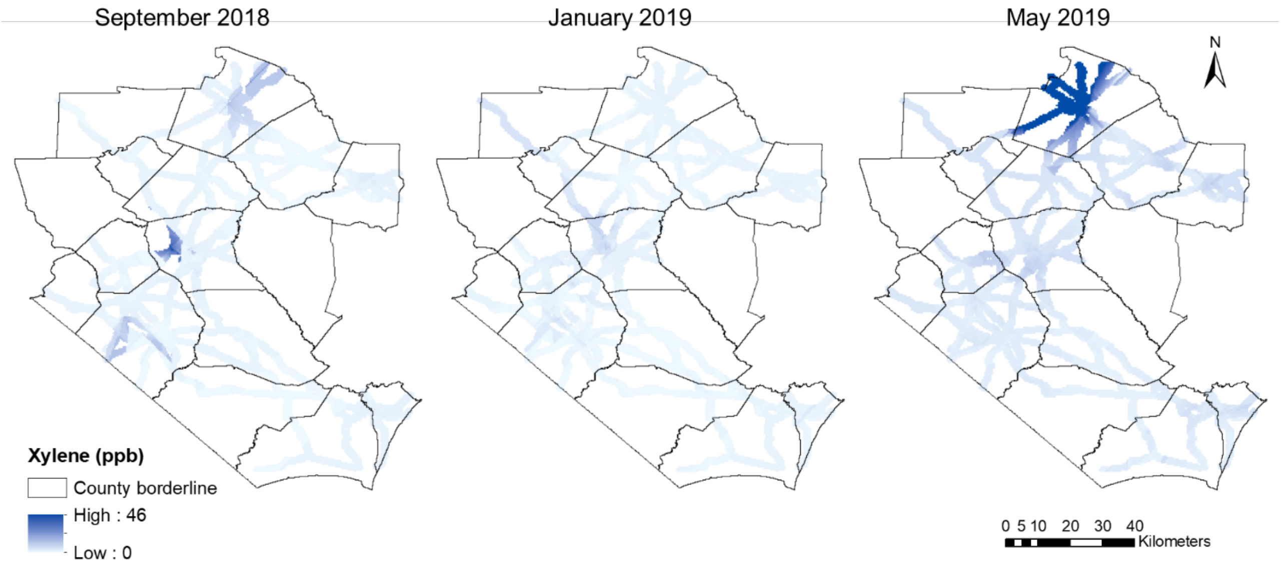

Figure S4. Spatial distribution of Xylene for September 2018, January 2019 and May 2019.

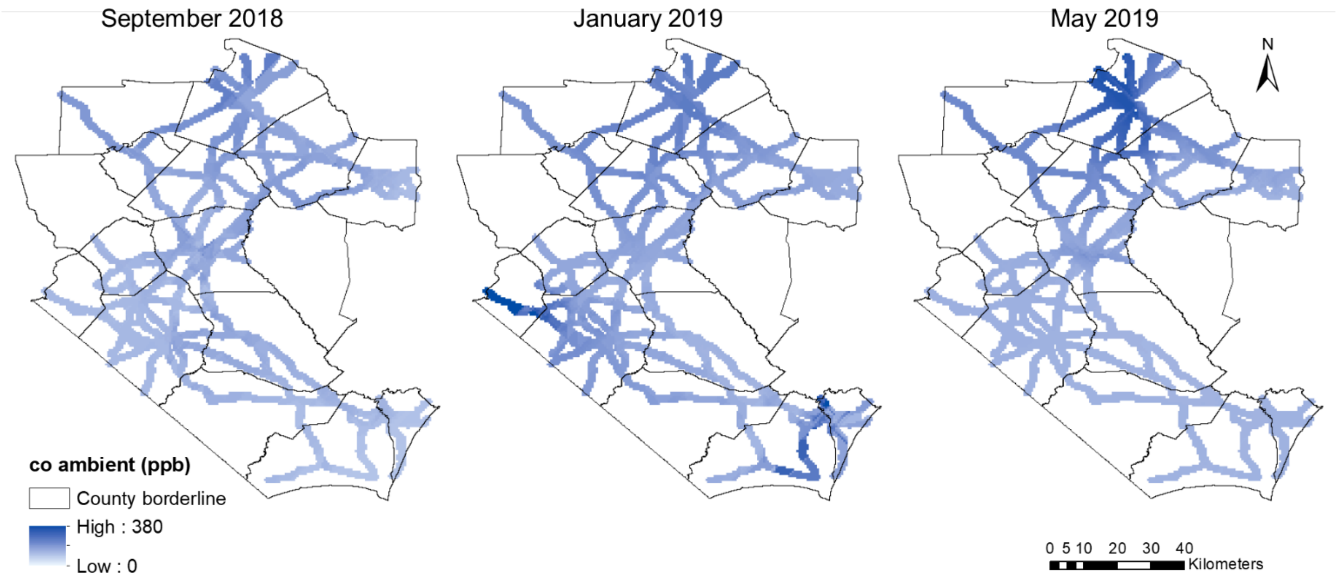

Figure S5. Spatial distribution of carbon monoxide (CO) for September 2018, January 2019 and May 2019.

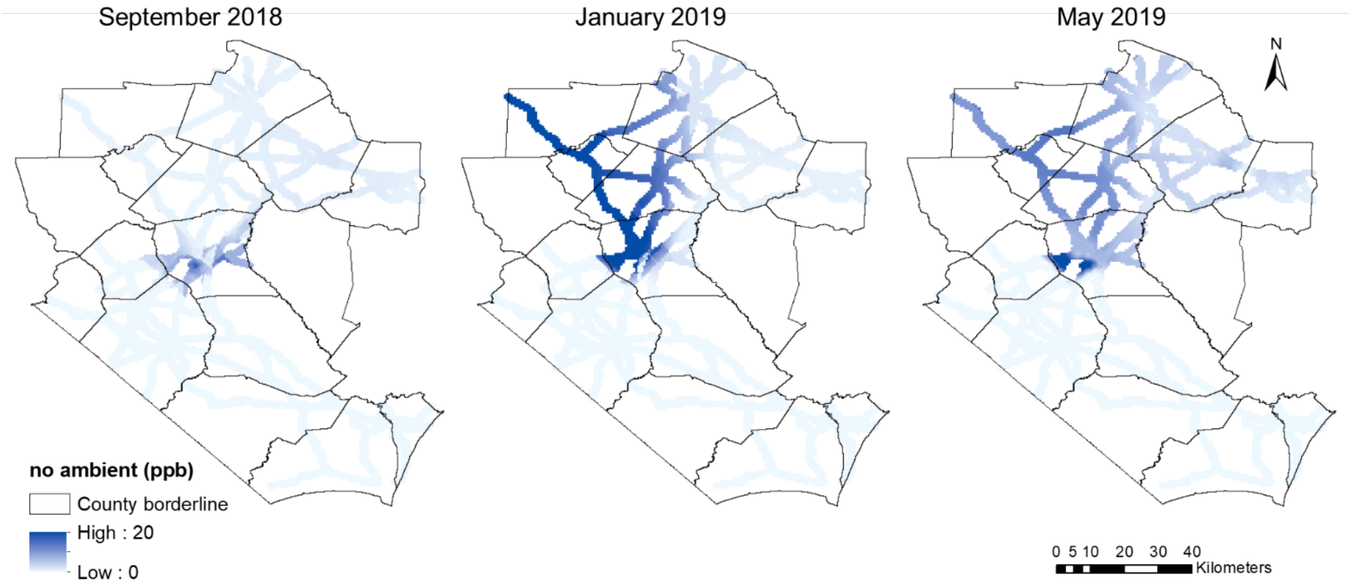

Figure S6. Spatial distribution of nitrogen monoxide (NO) for September 2018, January 2019 and May 2019.

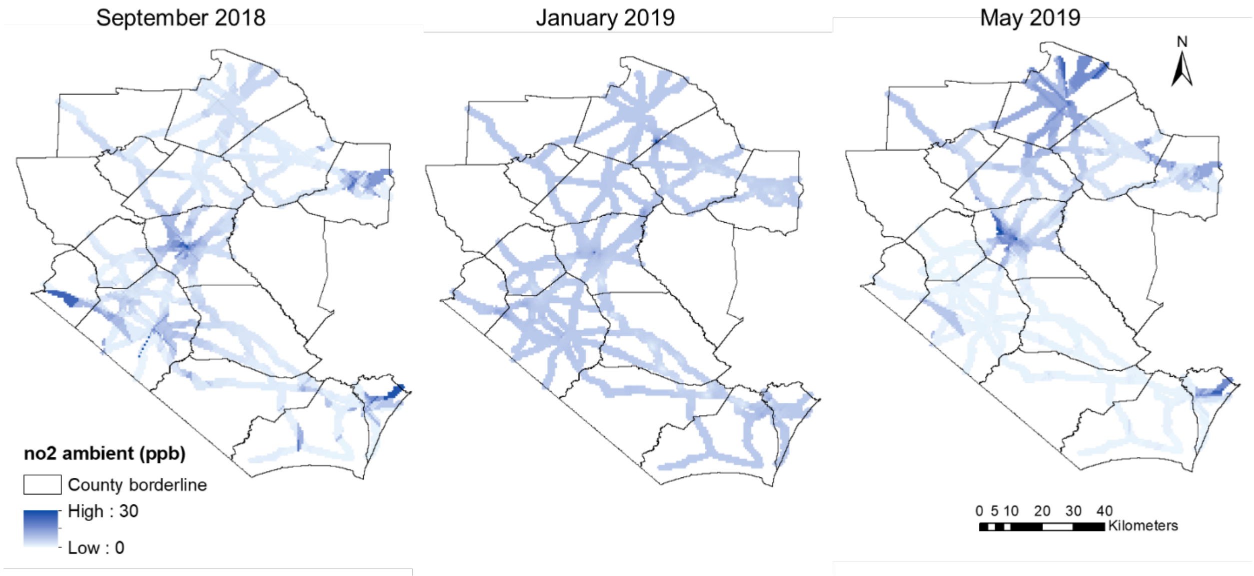

Figure S7. Spatial distribution of nitrogen dioxide (NO2) for September 2018, January 2019 and May 2019.

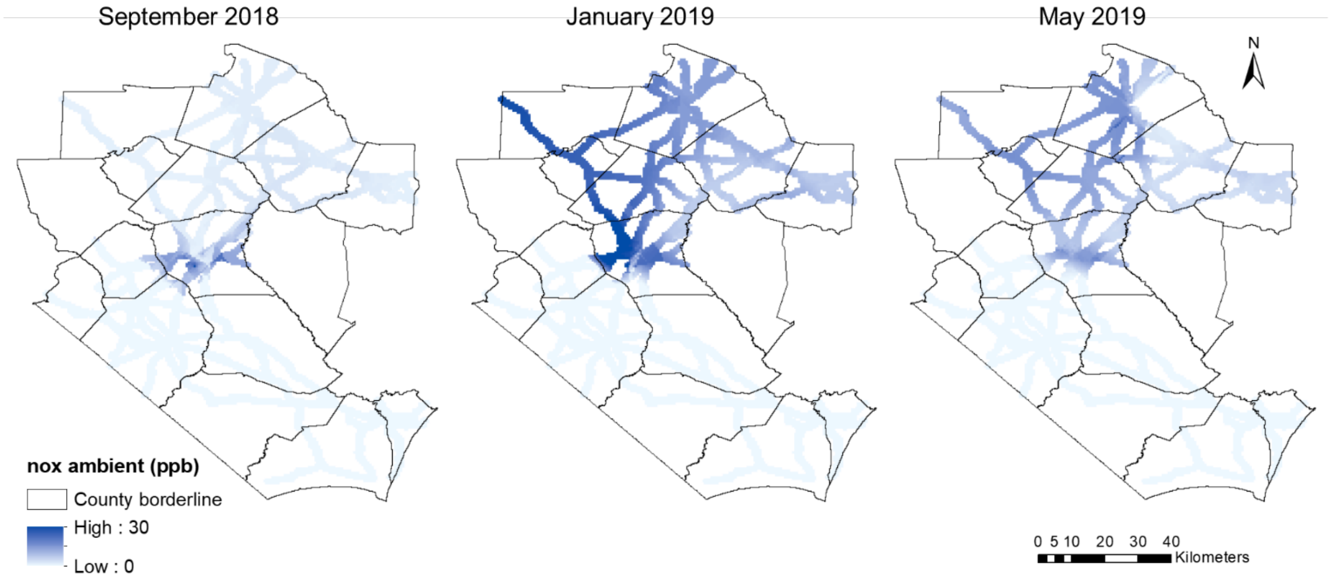

Figure S8. Spatial distribution of nitrogen oxides (NOx) for September 2018, January 2019 and May 2019.

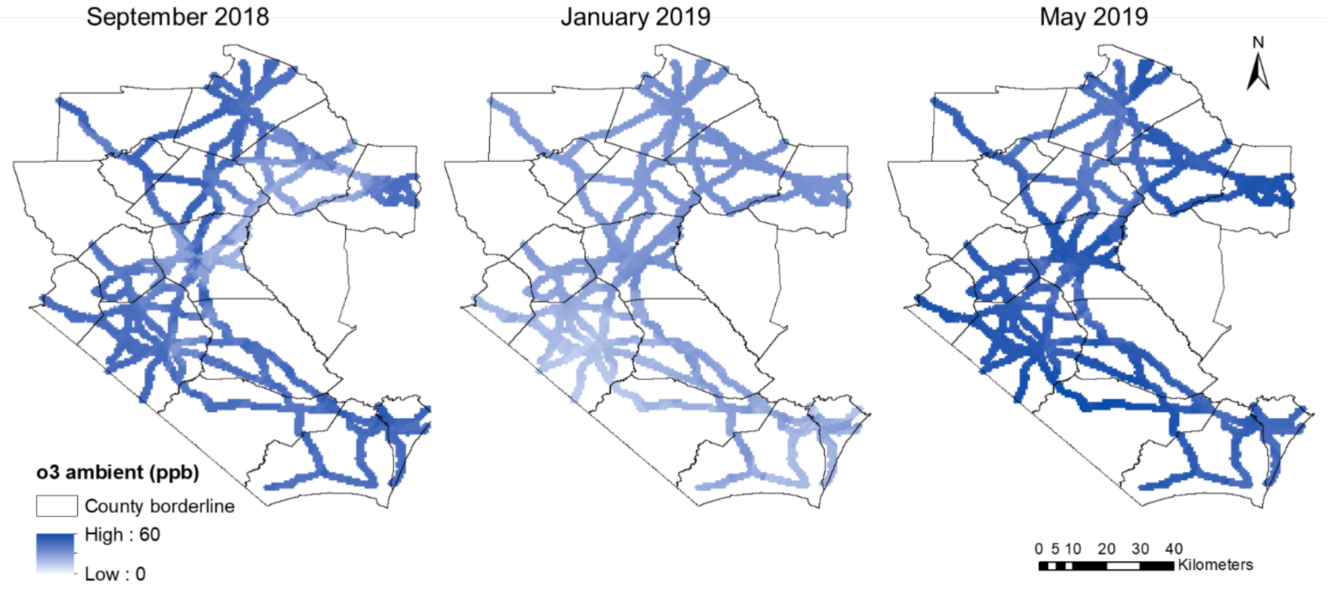

Figure S9. Spatial distribution of ozone (O3) for September 2018, January 2019 and May 2019.

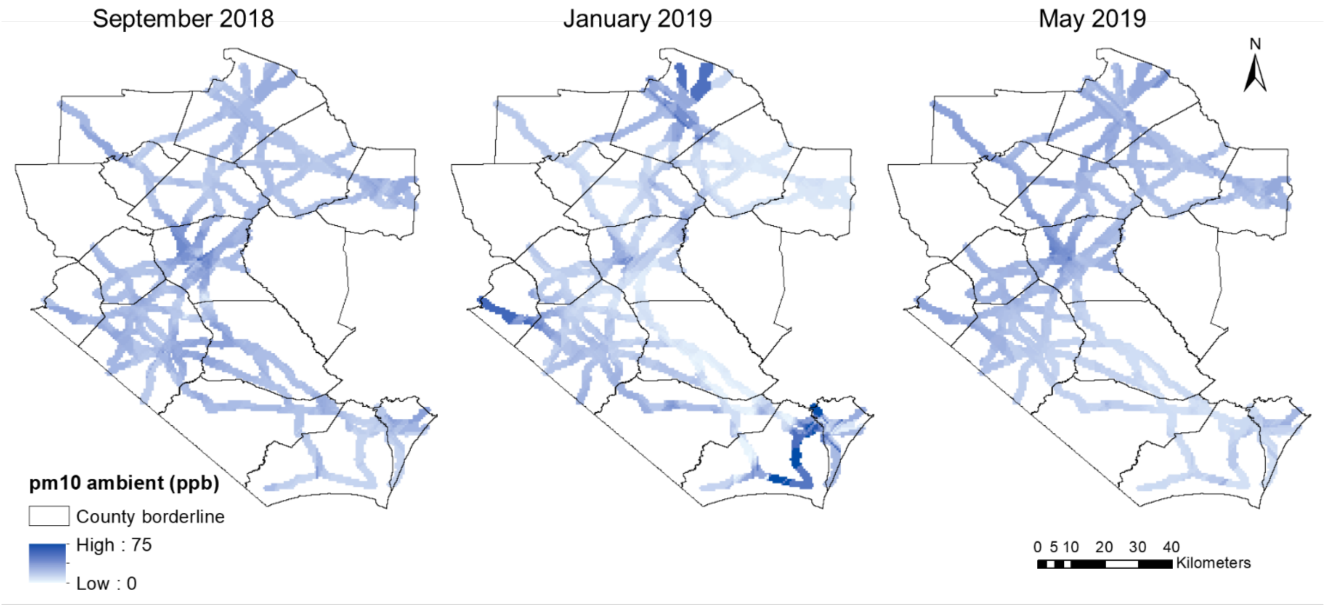

Figure S10. Spatial distribution of PM10 for September 2018, January 2019 and May 2019.

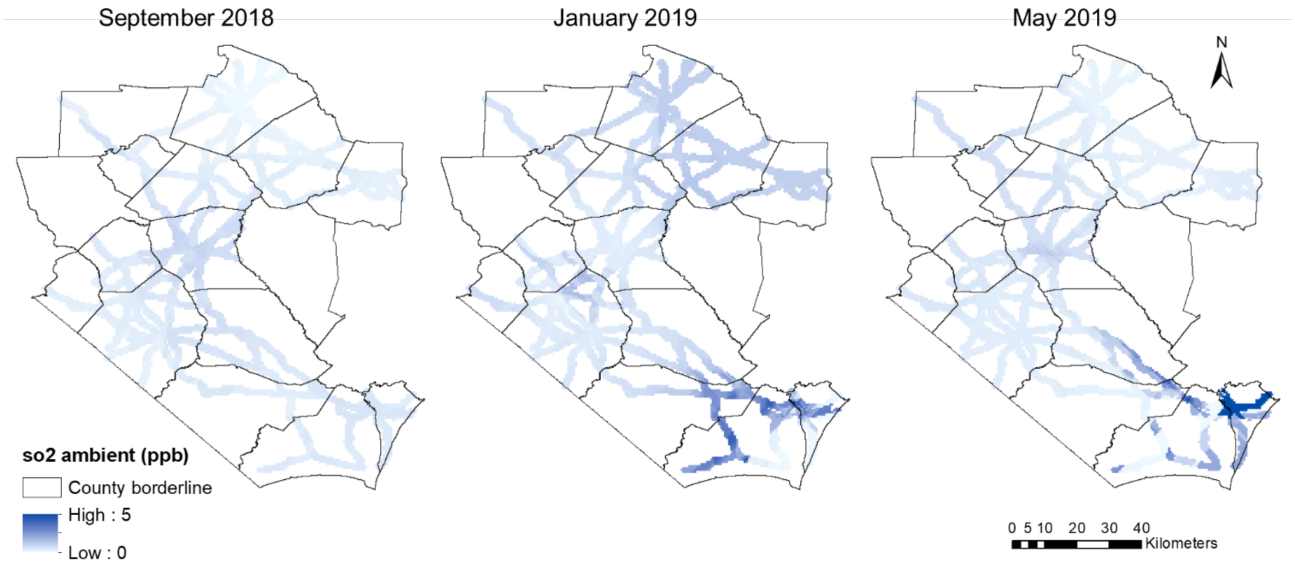

Figure S11. Spatial distribution of sulfur dioxide (SO2) for September 2018, January 2019 and May 2019.

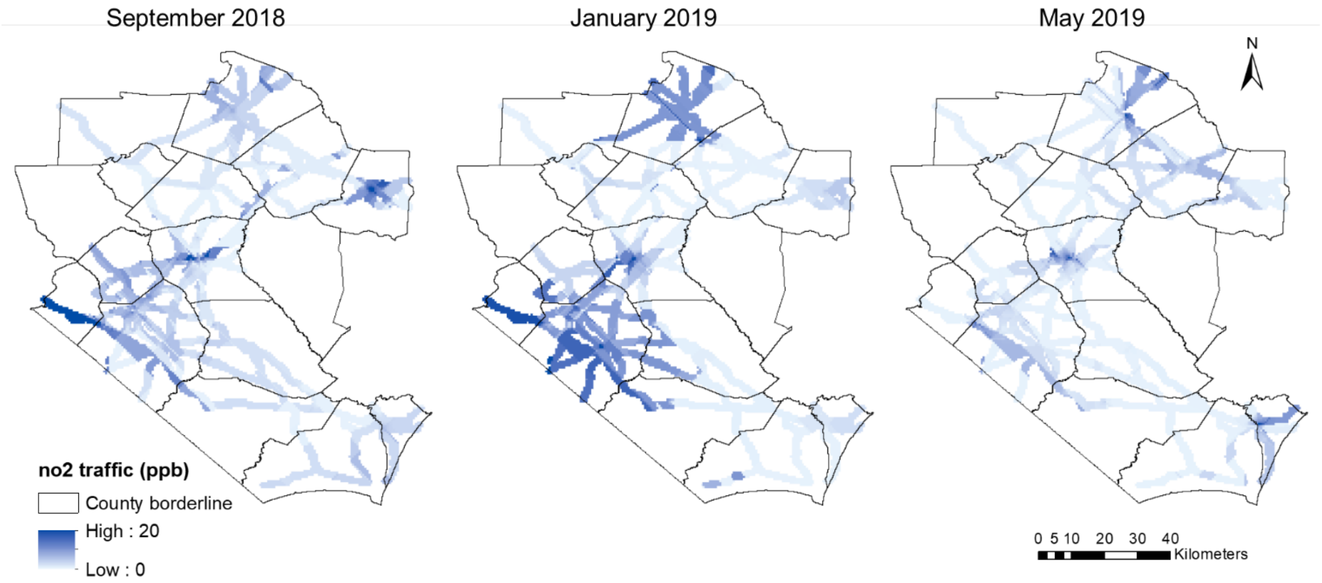

Figure S12. Spatial distribution of traffic-related nitrogen dioxide for September 2018, January 2019 and May 2019.

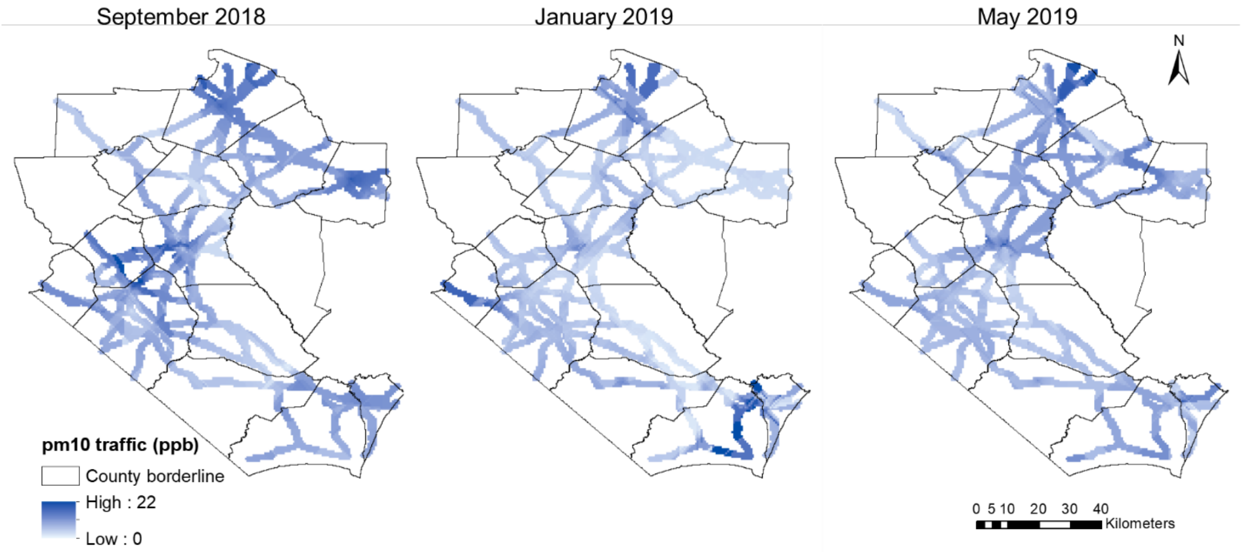

Figure S13. Spatial distribution of traffic-related PM10 for September 2018, January 2019 and May 2019.

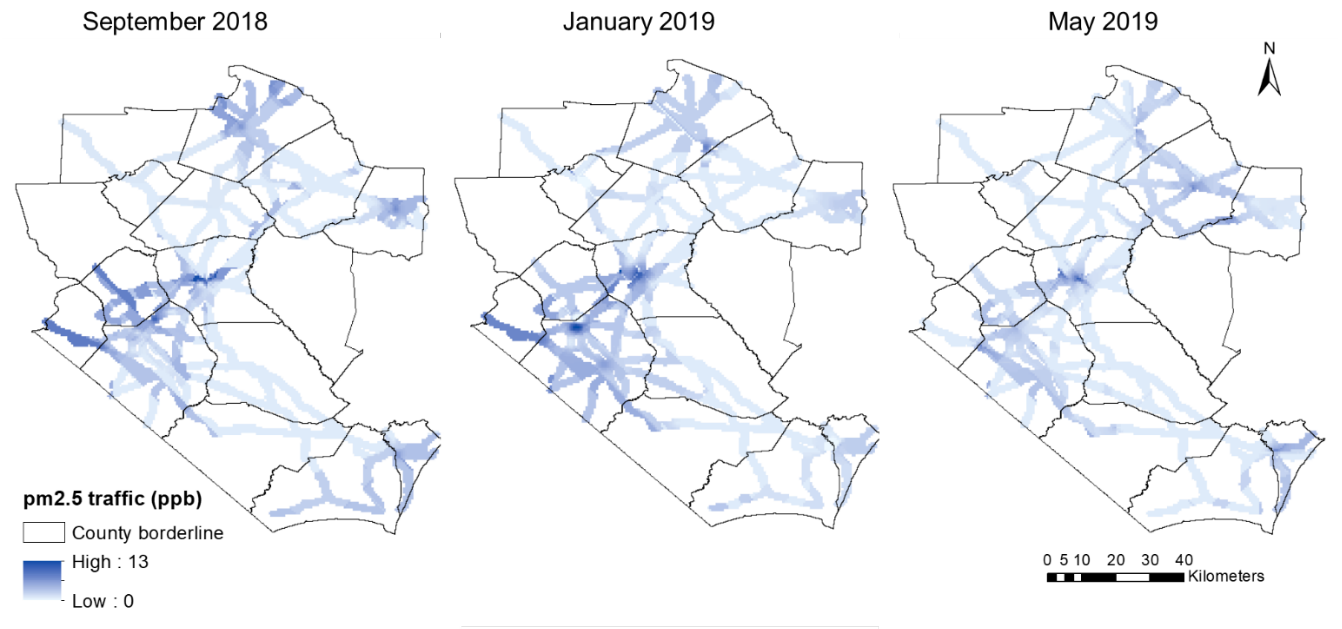

Figure S14. Spatial distribution of traffic-related PM2.5 for September 2018, January 2019 and May 2019.

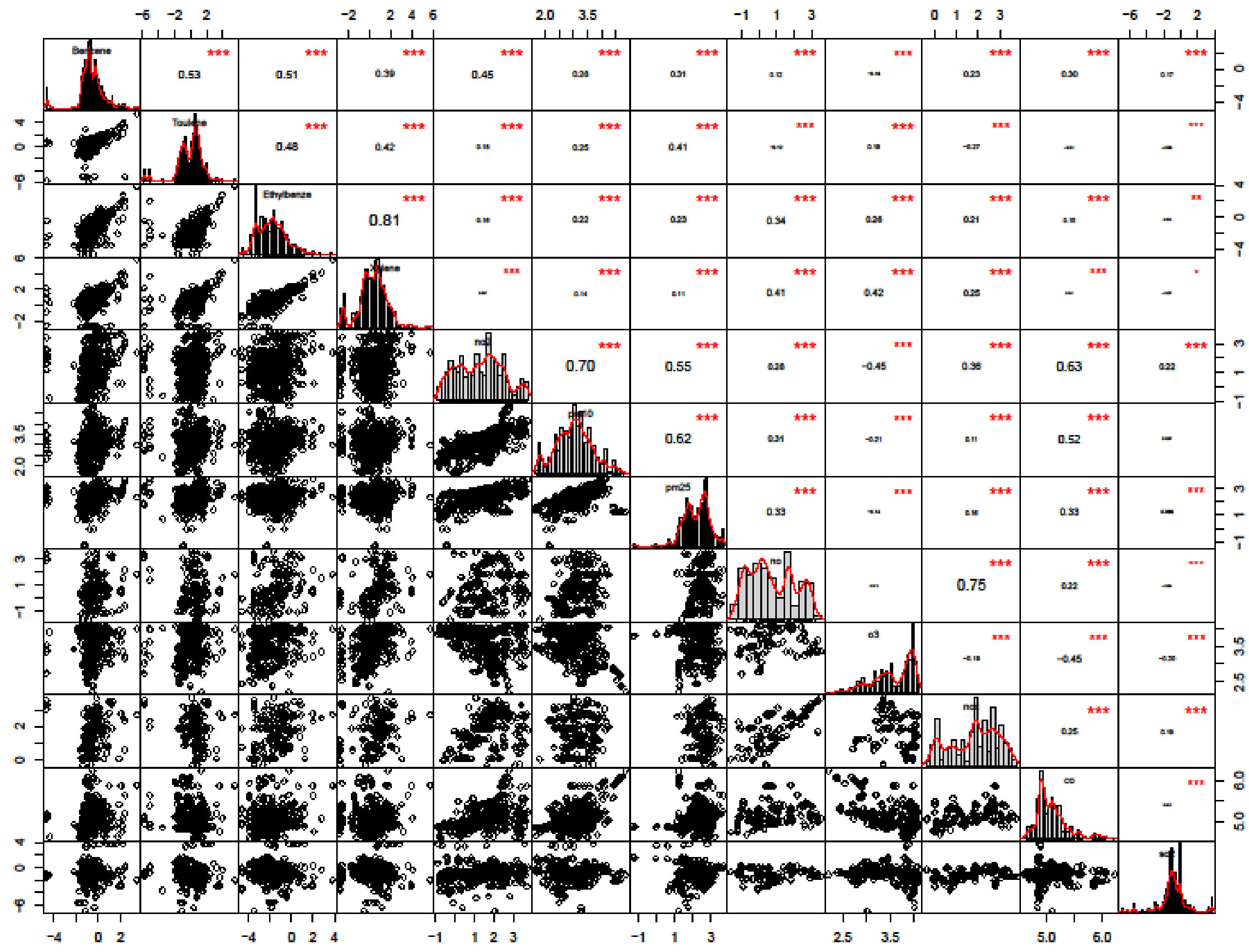

Figure S15. The scatter-matrix, and correlation matrix for measured BTEX with modeled NO2, PM10, PM25, NO, O3, NOx, CO, SO2.

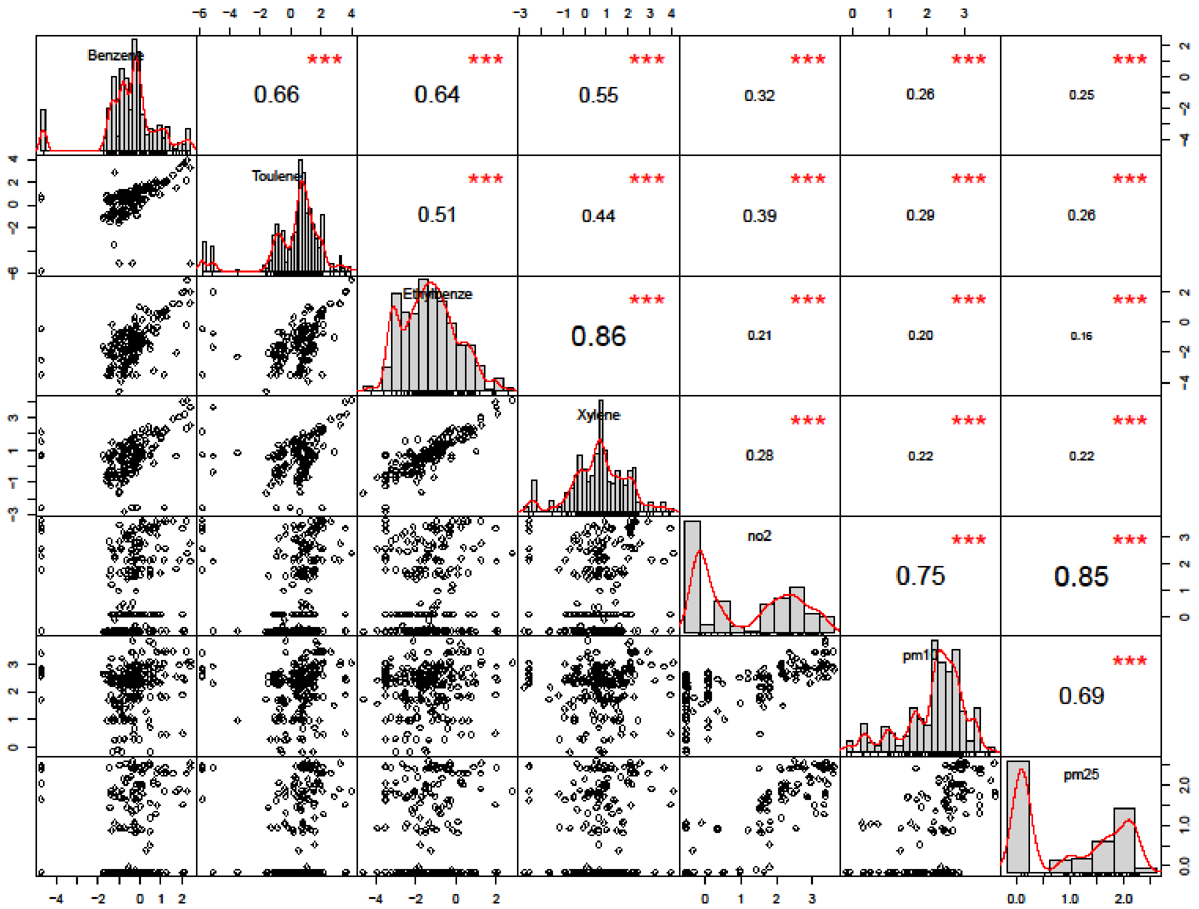

Figure S16. The scatter-matrix, and correlation matrix for measured BTEX with modeled traffic-related NO2, PM10 and PM2.5.
